# High temperatures affect the hypersensitive reaction, disease resistance and gene expression induced by a novel harpin HpaG-Xcm

**DOI:** 10.1038/s41598-018-37886-9

**Published:** 2019-01-30

**Authors:** Xiaoyun Zhou, Yue Liu, Jiamin Huang, Qinghuan Liu, Jianzhang Sun, Xinfeng Cai, Peng Tang, Wenbo Liu, Weiguo Miao

**Affiliations:** 1 Institute of Tropical Agriculture and Forestry, Hainan University, Haikou, Hainan Province China; 2 Key Laboratory of Green Prevention and Control of Tropical Plant Diseases and Pests (Hainan University), Ministry of Education, Haikou, 570228 Hainan Province China

## Abstract

Harpin proteins are produced by plant-pathogenic Gram-negative bacteria and regulate bacterial pathogenicity by inducing plant growth and defence responses in non-hosts. HpaG-Xcm, a novel harpin protein, was identified from *Xanthomonas citri* pv. *mangiferaeindicae*, which causes bacterial black spot of mango. Here, we describe the predicted structure and functions of HpaG-Xcm and investigate the mechanism of heat resistance. The HpaG-Xcm amino acid sequence contains seven motifs and two α-helices, in the N- and C-terminals, respectively. The N-terminal α-helical region contains two heptads, which form the coiled-coil (CC) structure. The CC region, which is on the surface of HpaG-Xcm, forms oligomeric aggregates by forming hydrophobic interactions between hydrophobic amino acids. Like other harpins, HpaG-Xcm was heat stable, promoted root growth and induced a hypersensitive response (HR) and systemic acquired resistance in non-host plants. Subjecting HpaG-Xcm to high temperatures altered the gene expression induced by HpaG-Xcm in tobacco leaves, probably due to changes in the spatial structure of HpaG-Xcm. Phenotypic tests revealed that the high-temperature treatments reduced the HR and disease resistance induced by HpaG-Xcm but had little effect on growth promotion. These findings indicate that the stability of interactions between CC and plants may be associated with thermal stability of HpaG-Xcm.

## Introduction

In recent years, bacterial black spot of mango, which can infect leaves, branches and fruits has become increasingly serious in the main mango-producing areas, severely impacting product quality and yield. The pathogen causing bacterial black spot of mango is a Gram-negative phytopathogenic bacteria^1^, which was first reported as ‘*Bacillus mangiferae*’^2^. In 1948, it was reclassified as ‘*Pseudomonas mangiferae-indicae*’^3^. In 1970, it was renamed ‘*Xanthomonas campestris* pv. *mangiferaeindicae*’^4^ according to the International Society for Plant Pathology’s international standards for naming pathovars of phytopathogenic bacteria. In 2009, Ah-You *et al*.^4^ reclassified the pathogen as “*Xanthomonas citri* pv. *mangiferaeindicae*” using AFLP (amplified fragment length polymorphism), MLSA (multilocus sequence analysis) and DNA molecular hybridization techniques.

Most plant pathogenic bacteria have two types of genes that regulate their interactions with plants. One is an avirulence gene (*avr*), which determines host specialization, and the other is an *hrp* (hypersensitive reaction and pathogenicity) gene, which is involved in pathogenicity on hosts and produces the hypersensitive reaction on non-hosts or resistant varieties^5^. *hrp* gene clusters have been found in *Pseudomonas syringae*, *P*. *syringae* pv. *phaseolicola*, *Erwinia amylovora*, *Ralstonia solanacearum* and *Xanthomonas campestris*^6^. Harpin proteins encoded by *hrp* genes are secreted by bacterial type III protein secretion systems (T3SS) and can stimulate a hypersensitive response (HR) on non-host plants^7^. The HR involves rapid local tissue and cell death induced by incompatible pathogens and promotes plant defence against a variety of bacteria, fungi, nematodes and viruses, which contributes to preventing further infection of the host by the pathogen. In addition to the HR, harpins have many common characteristics such as being heat-stable, glycine-rich and cysteine-deficient, and insensitive to acids and bases but sensitive to protease K and ultraviolet rays. To date, harpin proteins have been cloned from *Erwinia*, *Pseudomonas*, *Ralstonia* and *Xanthomonas*. Many harpin proteins from *Xanthomonas* spp. have been reported from different hosts, including HpaG-Xag from *X*. *axonopodis* pv. *glycines*, Hpa1Xac from *X*. *axonopodis* pv. *citri*, HpaXm from *X*. *citri* subsp. *malvacearum*, Hpa1Xoc from *X*. *oryzae* pv. *oryzicola*, Hpa1Xoo-2 from *X*. *oryzae* pv. *oryzae*, Hpa1Xoo from *X*. *oryzae* pv. *oryzae*, XopA-Xcv from *X*. *campestris* pv. *vesicatoria* and HpaXcc from *X*. *campestris* pv. *campestris*^5^. However, a harpin from *X*. *citri* pv. *mangiferaeindicae*, which is parasitic on tropical crops, has not previously been reported.

The amino acid sequences of harpin proteins from different plant pathogenic bacteria are different, whereas homologues of *Xanthomonas* spp. harpins from different species are highly homologous^5,8,9^. The molecular weights of harpins encoded by *Xanthomonas hpa1/hpaG* differ greatly from those of *Erwinia* and *Pseudomonas* harpins. The most significant difference between Hpa1 and HpaG is whether the protein N-terminal contains cysteine (Cys) residues^8^. The N-terminal α-helical regions of Hpa1Xoo and Hpa1Xoc contain one Cys each, whereas the Cys sites of HpaG-Xag, HpaXac and HpaXm are placed by a threonine (Thr) residue^6^. Both of the *Xanthomonas* spp. harpins contain two main α-helices, at the N- and C-terminals, respectively. The α-helical regions of N-terminals are necessary to induce HR in tobacco leaves^10^. Harpins are able to stimulate hypersensitive cell death (HCD) and systemic acquired resistance (SAR), and contribute to drought resistance, insect resistance and plant growth^7,11,12^. Previous studies^13^ have shown that the expression levels of genes associated with the defence response were significantly increased in harpin-treated plants.

Harpins have a strong thermal stability; however, the mechanism of heat resistance has been rarely reported. A study of HpaXm^6^ showed that the polypeptide fragment, which has only 14 amino acids, also had heat resistance. Disulfide bonds can form between cysteine residues. In view of the lack of cysteine in most harpins, Choi *et al*.^14^ suggested that the thermal stability of harpins might be due to the lack of cysteine, resulting in the absence of the tertiary structures formed by disulfide bonds. Tarafdar *et al*.^15^ pointed out that the coiled-coil (CC) motifs of harpins existed in the helical regions. Given that different aggregates seemed to be formed by the interactions between them, Tarafdar *et al*.^15^ speculated that the natural polymerization state of harpins might be related to their thermal stability.

In this study, we first describe the novel harpin HpaG-Xcm from *X*. *citri* pv. *mangiferae* and show that it has the highest homology with HpaG-Xag, which perfected the evolutionary relationship between harpin proteins from *Xanthomonas* spp. We also demonstrate that, like other harpins, HpaG-Xcm was a heat-stable protein and could induce HR and TMV resistance in tobacco leaves, promote the germination of *Arabidopsis thaliana* seeds, and enhance the expression levels of defence-related genes in tobacco leaves. In addition, we investigated the effect of high temperatures on HpaG-Xcm and showed that the HR activity, TMV resistance and expression levels of related genes induced by HpaG-Xcm on non-host plants changed, depending on the high-temperature treatment, which might be due to changes in the spatial structure of HpaG-Xcm. We also explored the effect of high temperature on harpin protein function from a micro perspective, so as to lay a foundation for exploring the heat-resistance mechanism of harpins. Research and development of the beneficial characteristics of HpaG-Xcm in inducing disease resistance and promoting growth should be helpful for disease control and increasing yields. Furthermore, given that HpaG-Xcm has a stronger growth-promoting effect than Hpa1Xoo, it should be more valuable than Hpa1Xoo in terms of agricultural development and application.

## Results

### Comparison of HpaG-Xcm sequence characteristics and nine homologues

**Figure 1:**
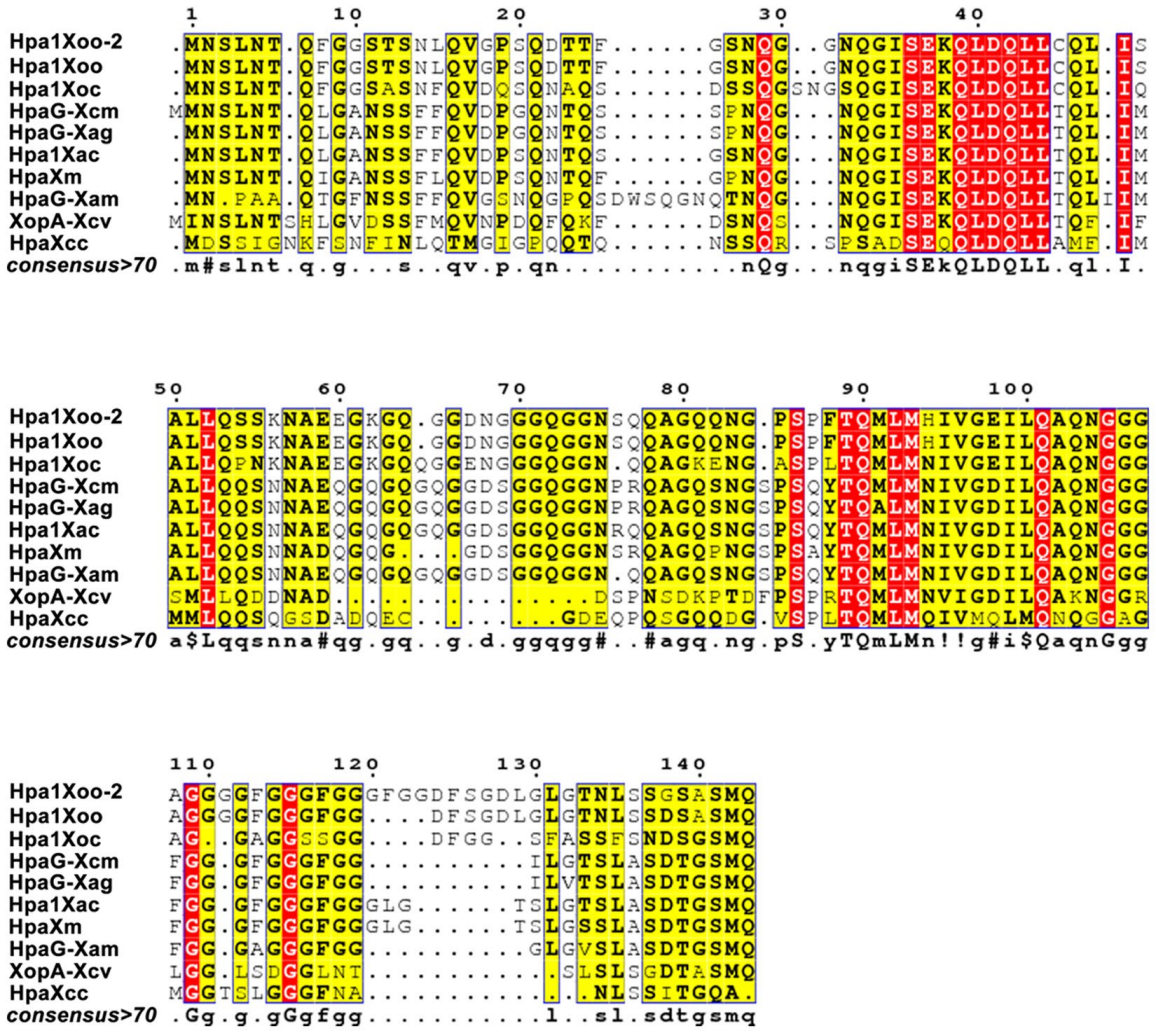
The multiple alignment of protein sequences of HpaG-Xcm and nine homologues. Multiple alignment was performed using ESPript 3.0 (http://espript.ibcp.fr/ESPript/cgi-bin/ESPript.cgi). The GenBank accession numbers of the 10 harpins are as follows: ATB17312.1 for HpaG-Xcm from *Xanthomonas citri* pv. *mangiferaeindicae*; ABK51582.1 for HpaG-Xag from *X*. *axonopodis* pv. *glycines*; ATB17313.1 for HpaG-Xam from *X*. *phaseoli* pv. *manihotis*; AAM35307.1 for Hpa1Xac from *X*. *axonopodis* pv. *citri*; ABG36696.1 for HpaXm from *X*. *citri* subsp. *malvacearum*; AAW59494.2 for Hpa1Xoc from *X*. *oryzae* pv. *oryzicola*; ABU48601.1 for Hpa1Xoo-2 from *X*. *oryzae* pv. *oryzae*; ACD56757.1 for Hpa1Xoo from *X*. *oryzae* pv. *oryzae*; AAL78294.1 for XopA-Xcv from *X*. *campestris* pv. *vesicatoria*; NP_636614.1 for HpaXcc from *X*. *campestris* pv. *campestris*.

We successfully amplified a 405 bp fragment with a GC content of 60.49% from the DNA genome of *Xanthomonas citri* pv. *mangiferaeindicae* HNHK strain, which was named *hpaG-Xcm* (KY697778.1). HpaG-Xcm (ATB17312.1), encoded by *hpaG-Xcm*, is composed of 134 amino acids, and the molecular mass was estimated to be 13.56 kDa. This protein was rich in glycine (21.6%) but lacking tryptophan and cysteine. A multiple sequence alignment of HpaG-Xcm and nine amino acid sequences of homologous harpin proteins from *Xanthomonas* spp. was carried out (Fig. 1). The homologies between HpaG-Xcm and HpaG-Xam (ATB17313.1) from *X*. *phaseoli* pv. *manihotis*, Hpa1Xoo-2 (ABU48601.1) from *X*. *oryzae* pv. *oryzae*, Hpa1Xoo (ACD56757.1) from *X*. *oryzae* pv. *oryzae*, HpaXm (ABG36696.1) from *X*. *citri* subsp. *malvacearum*, Hpa1Xoc (AAW59494.2) from *X*. *oryzae* pv. *oryzicola*, XopA-Xcv (AAL78294.1) from *X*. *campestris* pv. *vesicatoria*, HpaXcc (NP_636614.1) from *X*. *campestris* pv. *campestris*, HpaG-Xag (ABK51582.1) from *X*. *axonopodis* pv. *glycines* and Hpa1Xac (AAM35307.1) from *X*. *axonopodis* pv. *citri* were 87.8%, 71.8%, 72.5%, 91.5%, 66.9%, 56.9%, 39%, 98.5% and 95.5%, respectively.

**Figure 2:**
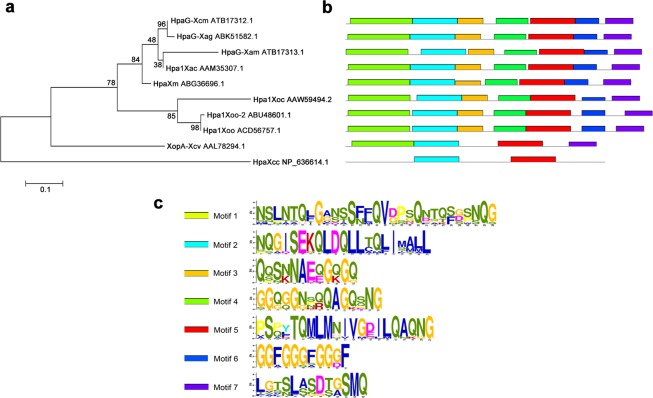
Phylogenetic tree of complete amino acid sequences and conservative analysis for characteristic domains of HpaG-Xcm and nine harpins from *Xanthomonas* spp. (**a**) Phylogenetic analysis of the complete amino acid sequences of HpaG-Xcm and nine harpins from *Xanthomonas* spp. using MEGA5.0 software analysis. The phylogenetic tree was constructed using the Maximum Likelihood method, and the Bootstrap value was set to 1000. HpaG-Xcm from *X*. *citri* pv. *mangiferaeindicae*, HpaG-Xag from *X*. *axonopodis* pv. *glycines*, HpaG-Xam from *X*. *phaseoli* pv. *manihotis*, Hpa1Xac from *X*. *axonopodis* pv. *citri*, HpaXm from *X*. *citri* subsp. *malvacearum*, Hpa1Xoc from *X*. *oryzae* pv. *oryzicola*, Hpa1Xoo-2 from *X*. *oryzae* pv. *oryzae*, Hpa1Xoo from *X*. *oryzae* pv. *oryzae*, XopA-Xcv from *X*. *campestris* pv. *vesicatoria*, HpaXcc from *X*. *campestris* pv. *campestris*. (**b**,**c**) Conservative analysis for characteristic domains of HpaG-Xcm and nine harpins from *Xanthomonas* spp. and their positions in sequences determined using MEME (http://meme-suite.org/tools/meme). The stacking height of amino acids at different sites of the sequence are shown as the conserved degree of the site, and the height of the single amino acid in the stack shows the relative frequency of this amino acid at this position.

A phylogenetic tree was constructed based on conservative sequence analysis of the complete amino acid sequences of HpaG-Xcm and the nine homologous harpin proteins from *Xanthomonas* spp. (Fig. 2a). Seven relatively conservative motifs that were arranged in the same order were identified in all 10 ten proteins r (Fig. 2b,c). The phylogenetic tree grouped the 10 proteins into three branches: HpaG-Xcm, HpaG-Xag, HpaG-Xam, Hpa1Xac and HpaXm; Hpa1Xoc, Hpa1Xoo-2 and Hpa1Xoo; and XopA-Xcv and HpaXcc (Fig. 2a). The shortest of the seven conserved motifs was composed of 11 amino acid residues, and the longest motif was composed of 29 amino acid residues (Fig. 2c). The motif characteristics of the homologous proteins were basically consistent with their position on the phylogenetic tree.

### Secondary structure, tertiary structure and coiled-coil predictions of HpaG-Xcm

**Figure 3:**
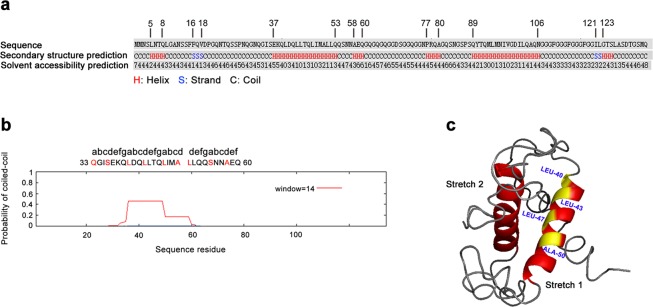
Structure prediction analyses of HpaG-Xcm. (**a**) Secondary structure and residue solvent accessibility predictions for HpaG-Xcm using I-TASSER (https://zhanglab.ccmb.med.umich.edu/I-TASSER/). Values range from 0 (buried residue) to 9 (highly exposed residue). (**b**) Coiled coil (CC) prediction of HpaG-Xcm. The CC motif was predicted using Coiled-Coil Prediction (http://npsa-pbil.ibcp.fr/cgi-bin/npsa_automat.pl?page=npsa_lupas.html). The letters *a* to *g* designate positions in the heptad repeat. The CC-forming probability was obtained by scanning windows of 14 residues with the MTIDK matrix. Amino acid residues located at *a* and *d* are marked in red. (**c**) Ribbon representation of the 3D structure of HpaG-Xcm. α-helical motifs are highlighted in red. Residues involved in the coiled-coil motif are highlighted in yellow. (Stretch 1) is a predicted α-helix in the N-terminal region; (Stretch 2) is a predicted α-helix in the C-terminal region. The tertiary structure of HpaG-Xcm was predicted by I-TASSER (https://zhanglab.ccmb.med.umich.edu/I-TASSER/). The 3D structure model was modified using PyLOM software.

Structure prediction analyses indicated that the secondary structure of HpaG-Xcm consists of helixes, strands and coils. There is an α-helix at the N-terminal and C-terminal of HpaG-Xcm, which have the sequences 37-EKQLDQLLTQLIMALLQ-53 and 89-QYTQMLMNIVGDILQAQN-106, respectively (Fig. 3a). The solvent accessibility prediction of amino acid residues showed that the values of Leu40, Leu44, Ile48, Met93, Leu94 and Val98 were 0 (Fig. 3a), indicating that these amino acid residues were located within the structure. Furthermore, these amino acids were distributed in the α-helix regions.

The coiled-coil (CC) prediction indicated that Gln33 to Gln60 of the HpaG-Xcm amino acid sequence had the possibility of forming a CC, and that there were two complete heptads at the N-terminal: 33-QGISEKQ-LDQLLTQ-46 (Fig. 3b). The 3D model of HpaG-Xcm showed that the structure of this protein was predominantly made up of helical regions connected by turns and loops (Fig. 3c). Leu40, Leu43, Leu47 and Ala50 are critical amino acids for the formation of the CC structure (Fig. 3b), and are located on the hydrophobic side of the α-helix (Fig. 3c).

### Expression and purification of HpaXcm

In order to explore the optimum expression conditions for GST-HpaXcm (the fusion protein GST-HpaG-Xcm), we investigated the effect of different isopropyl-β-D-thiogalactoside (IPTG) concentrations (0.05 mM and 0.1 mM), different induction times (3 h and 5 h) and different induction temperatures (28 °C and 37 °C) on the expression of the fusion protein. SDS-PAGE analysis indicated that GST-HpaXcm was expressed as two forms: soluble proteins and inclusion bodies (Supplemental Fig. S1). Although the GST-HpaXcm fusion protein (39 kDa) could be expressed under different conditions, different induction conditions led to a different proportion of soluble protein in the total protein. When the expressed fusion soluble proteins were infiltrated into the intercellular space of tobacco leaves, all the proteins induced by the different conditions were able to induce HR production in tobacco leaves; however, the necrosis ratios (i.e., the ratio of necrotic area to injection area) were different. The maximum necrosis ratio was close to 1, which was induced by soluble protein C (Supplemental Fig. S1). Therefore, the optimum expression conditions for GST-HpaXcm is an IPTG concentration of 0.1 mM and 5 h of induction at 28 °C.

SDS-PAGE and a western blot were performed using a purified sample of the GST-HpaXcm fusion protein. A band of GST-HpaXcm appeared at 39 kDa, which was consistent with the theoretical molecular weight (the molecular masses of the GST-tag protein and HpaXcm are 26 kDa and 13.56 kDa, respectively) (Supplemental Fig. S2). GST-HpaXcm was digested by thrombin to obtain the GST-tag protein and the purified HpaXcm (Supplemental Fig. S2).

### Induced HR, disease resistance and root growth promotion by HpaXcm

**Figure 4:**
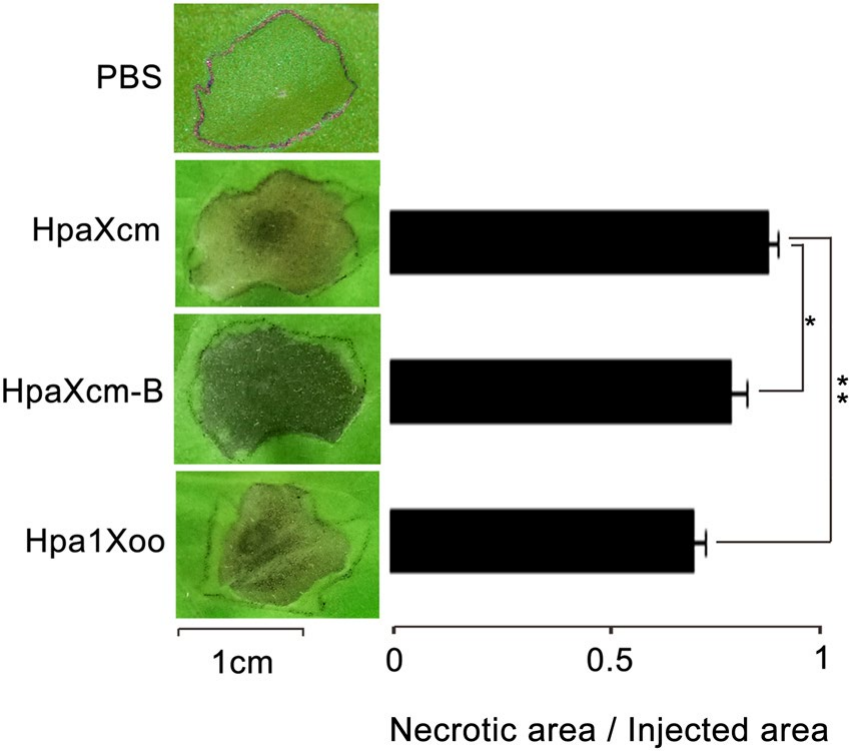
HR elicited by purified HpaXcm, purified HpaXcm-B (purified protein HpaG-Xcm boiled), purified Hpa1Xoo. (Left) Tobacco leaves were injected with the proteins at a concentration of 10 μM. Necrotic lesions were observed after 2 days. PBS was used as a negative control. The scale bar represents 1 cm. (Right) The histogram expresses the ratio of the necrotic area to the injected area. Error bars indicate the standard error of the mean (n = 3). Asterisks indicate a significant difference obtained by one-way ANOVA and multiple comparisons (LSD method, *P < 0.05, **P < 0.01). The experiment was repeated three times with the same result.

To determine whether HpaXcm could induce an HR response in tobacco leaves, purified HpaXcm (10 μM), purified and boiled HpaXcm-B (10 μM) or purified Hpa1Xoo (10 μM) were injected into tobacco leaves; phosphate-buffered saline (PBS) was used as a negative control. Unlike the negative controls, HpaXcm, HpaXcm-B and Hpa1Xoo induced a visible HR in the tobacco leaves (Fig. 4 left). The ratio of the necrotic area to the injected area of HpaXcm was significantly greater than that of HpaXcm-B (*P* < 0.05) and Hpa1Xoo (*P* < 0.01) (Fig. 4 right)

**Figure 5:**
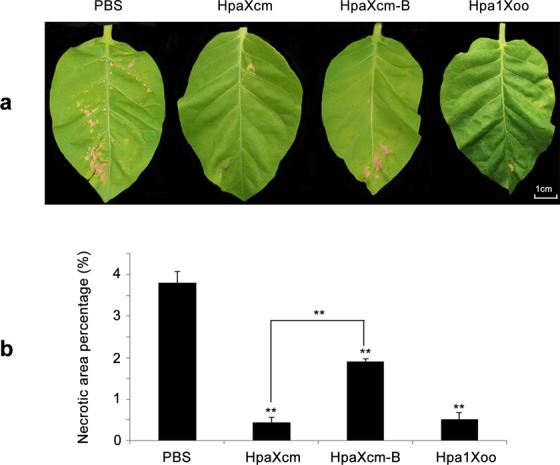
Disease resistance of tobacco to TMV that has been pretreated with purified HpaXcm, purified HpaXcm-B or purified Hpa1Xoo. (**a**) Tobacco leaves were pretreated with 10 μM purified HpaXcm, purified HpaXcm-B or purified Hpa1Xoo 16 h before inoculation with TMV. PBS was used as a negative control. The scale bar represents 1 cm. (**b**) Ratio of lesion area to tobacco leaf area. Tobacco leaves were inoculated with proteins at a concentration of 10 μM. PBS was used as a negative control. Necrotic lesions were observed 6 days after TMV inoculation. Error bars indicate the standard error of the mean (n = 3). Asterisks indicate a significant difference obtained by one-way ANOVA and multiple comparisons (LSD method, *P < 0.05, **P < 0.01). The experiment was repeated three times with the same result.

To determine whether HpaXcm could induce disease resistance, equal volumes of HpaXcm, HpaXcm-B, Hpa1Xoo or PBS (which was used as a negative control) were sprayed on tobacco leaves 16 h before inoculating the leaves with TMV. After 6 d, all the tobacco leaves had developed necrotic spots (Fig. 5a); however, the proportion of the leaf area that was necrotic was significantly smaller (*P* < 0.01) for leaves that had been treated with HpaXcm, HpaXcm-B or Hpa1Xoo compared with that of the control group. The necrotic area of leaves treated with HpaXcm-B was greater (*P* < 0.01) than that of leaves treated with HpaXcm; however, the necrotic areas of leaves treated with HpaXcm or Hpa1Xoo were not significantly different (Fig. 5b).

**Figure 6:**
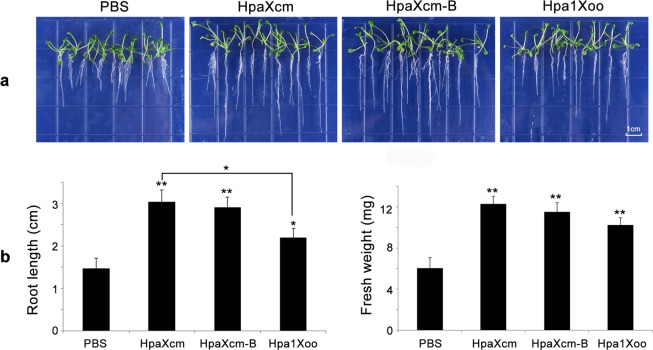
Effects of HpaXcm, HpaXcm-B and Hpa1Xoo on *Arabidopsis* root growth. (**a**) Germination of *A*. *thaliana* seeds soaked in purified HpaXcm, purified HpaXcm-B or purified Hpa1Xoo for 6 hours in advance and cultured on MS medium for 15 days. The scale bar represents 1 cm. (**b**) Quantification of root length and fresh weight 15 days after transfer to MS medium. Error bars indicate the standard error of the mean (n = 3). Asterisks indicate a significant difference obtained by one-way ANOVA and multiple comparisons (LSD method, *P < 0.05, **P < 0.01). The experiment was repeated three times with the same result.

To determine whether HpaXcm could promote root growth, *Arabidopsis thaliana* seeds were soaked in the same concentration of HpaXcm, HpaXcm-B, Hpa1Xoo or PBS (which was used as a negative control) for 6 h, before transferring the seeds to MS medium for culture. After 15 d, seeds soaked in HpaXcm, HpaXcm-B or Hpa1Xoo produced stronger roots and more branches than those in the control group (Fig. 6a). The mean root length and mean fresh weight were also significantly greater (*P* < 0.05) than those of the control group. The mean root length of *A*. *thaliana* plants that grew from seeds that had received the HpaXcm treatments were significantly greater (*P* < 0.05) than those that grew from seeds that had received the Hpa1Xoo treatment (Fig. 6b). However, the plants that grew from seeds that had received the HpaXcm or HpaXcm-B treatment were not significantly different (Fig. 6b).

### qRT-PCR

**Figure 7:**
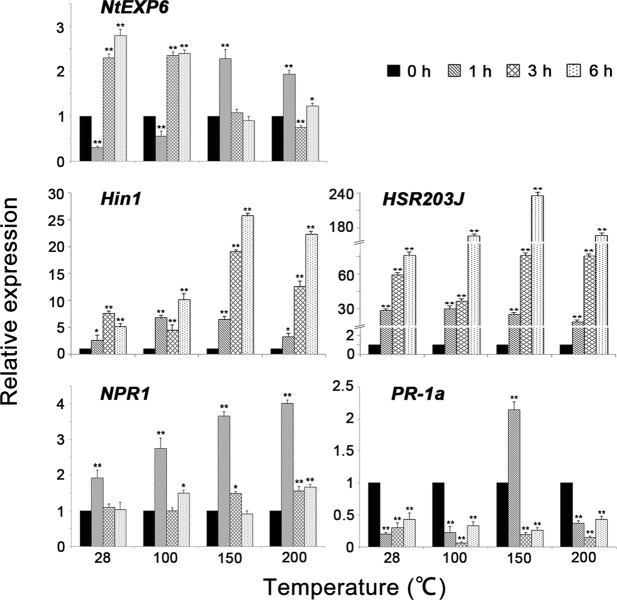
Expression levels of *NtEXP6*, *Hin1*, *HSR203J*, *NPR1* and *PR-1a* in tobacco leaves elicited by HpaXcm at different times (0 h, 1 h, 3 h and 6 h) after heat treatment at different temperatures (28 °C, 100 °C, 150 °C and 200 °C). Relative gene expression was calculated using the 2^−ΔΔCT^ formula with *EF-1a* as a control for normalization. The tobacco leaves were harvested individually at 0, 1, 3 and 6 h post spraying and frozen in liquid nitrogen. cDNA was reverse-transcribed from total RNA and subjected to a quantitative reverse-transcriptase polymerase chain reaction. Expression levels of *NtEXP6*, *Hin1*, *HSR203J*, *NPR1* and *PR-1a* were quantified using a standard curve of pooled cDNA samples. Error bars indicate the standard error of the mean (n = 3). Asterisks indicate a significant difference obtained by one-way ANOVA and multiple comparisons (LSD method, *P < 0.05, **P < 0.01). The experiment was repeated three times with the same result.

In order to further understand whether HpaXcm induces HR, disease resistance and growth promotion because related genes were stimulated to increase their expression levels, and to explore the effects of temperature on protein functions, we compared the relative expression levels of the HR-related genes *Hin1*^16^ and *HSR203J*^17^, the defence-related genes *NPR1* and *PR-1a*^11^, and the growth promotion-related gene *NtEXP6*^18^ in tobacco leaves at 1 h, 3 h and 6 h with the expression levels in tobacco leaves at 0 h by performing quantitative real-time PCR (qRT - PCR). The relative expression levels of genes were calculated using the 2^−ΔΔCT^ formula with *EF-1a* as a control for normalization. In general, the expression of *NtEXP6*, *Hin1*, *HSR203J* and *NPR1* was clearly detected in tobacco leaves within 6 h of being treated with unheated HpaXcm (28 °C HpaXcm). However, when leaves were injected with HpaXcm that had been heated at different temperatures, the relative expression levels of *NtEXP6*, *Hin1*, *HSR203J*, *NPR1* and *PR-1a* changed (Fig. 7).

#### NtEXP6

Leaves treated with unheated (28 °C) HpaXcm did not express *NtEXP6* 1 h after treatment; however, significant expression (*P* < 0.01) of *NtEXP6* was observed at 3 h and at 6 h. The highest levels of expression were observed at 6 h. Leaves treated with unheated (28 °C) HpaXcm induced higher levels of *NtEXP6* expression than those treated with HpaXcm that had been heated to 100 °C, 150 °C or 200 °C. The relative expression induced by 28 °C HpaXcm and 100 °C HpaXcm peaked at 6 h; however, the relative expression induced by 150 °C HpaXcm and 200 °C HpaXcm peaked at 1 h. The peak values of relative expression induced by HpaXcm treated at different temperatures ranging from high to low were: 28 °C HpaXcm, 100 °C HpaXcm, 150 °C HpaXcm and 200 °C HpaXcm (Fig. 7).

#### Hin1

Leaves treated with 28 °C HpaXcm showed significant (*P* < 0.05) relative expression of *Hin1* at 1 h, expression peaked at 3 h, and then declined; however, the relative expression was still remarkable compared with that observed at 0 h. Leaves treated with 100 °C HpaXcm, 150 °C HpaXcm or 200 °C HpaXcm showed significant expression levels of *Hin1* within 6 h of the treatment, with relative expression peaking at 6 h, unlike the leaves subjected to the 28 °C HpaXcm treatment. The peak values of relative expression induced by HpaXcm treated at different temperatures ranging from high to low were: 150 °C HpaXcm, 200 °C HpaXcm, 100 °C HpaXcm and 28 °C HpaXcm (Fig. 7).

#### HSR203J

*HSR203J* was significantly (*P* < 0.01) expressed in leaves within 6 h of treatment with 28 °C HpaXcm, 100 °C HpaXcm, 150 °C HpaXcm or 200 °C HpaXcm, and the peak times of relative expression were 6 h. The peak values of relative expression induced by HpaXcm treated at different temperatures ranging from high to low were: 150 °C HpaXcm, 200 °C HpaXcm, 100 °C HpaXcm and 28 °C HpaXcm (Fig. 7).

#### NPR1

*NPR1* was significantly (*P* < 0.01) expressed in leaves treated with 28 °C HpaXcm, 100 °C HpaXcm, 150 °C HpaXcm or 200 °C HpaXcm, with relative expression peaking at 1 h. Leaves treated with 28 °C HpaXcm showed hardly any expression at 3 h and 6 h. The 150 °C HpaXcm treatment induced *NPR1* expression at 1 h and 3 h but not at 6 h. The peak values of relative expression induced by HpaXcm treated at different temperatures ranging from high to low were: 200 °C HpaXcm, 150 °C HpaXcm, 100 °C HpaXcm and 28 °C HpaXcm (Fig. 7).

#### PR-1a

*PR-1a* expression was only induced in leaves treated with 150 °C HpaXcm at 1 h. No expression was induced in leaves treated with 28 °C HpaXcm, 100 °C HpaXcm, or 200 °C HpaXcm within 6 h (Fig. 7).

## Discussion

Phylogenetic analysis revealed that HpaXcm and the nine harpin proteins from *Xanthomonas* contained seven conservative motifs and could be divided into two classes: HpaG-Xcm, HpaG-Xag, HpaG-Xam, Hpa1Xac, HpaXm, Hpa1Xoc, Hpa1Xoo-2 and Hpa1Xoo; and XopA-Xcv and HpaXcc (Fig. 2). Conserved motifs are closely related to protein structure and function^19^. Seven motifs were found in all eight amino acid sequences of the first group, whereas only four and two motifs were found in XopA-Xcv and HpaXcc, respectively. Harpins can be classified as ‘T’ or ‘C based on the Thr residue and Cys residue in the N-terminal α-helix region. Based on their ‘T’ and ‘C’ type, the first group of *Xanthomonas* spp. harpins can be divided into two branches: HpaG-Xcm, HpaG-Xag, HpaG-Xam, Hpa1Xac and HpaXm belong to the ‘T’ type and Hpa1Xoc, Hpa1Xoo-2 and Hpa1Xoo belong to the ‘C’ type^6^. HpaXcm contains seven motifs in a complete sequence and a Thr residue in the N-terminal α-helix region. On the basis of the amino acid composition and the arrangements of motifs of the 10 harpins we investigated, HpaXcm showed the closest relationship to HpaG-Xag.

Previous studies have indicated that *Xanthomonas* spp. harpins generally have two α-helices located in the N- and C-terminal. The N-terminal α-helices are crucial for inducing the HR of tobacco leaves^14^, and the two heptads in the α-helical regions influence the HR activity of proteins^10^. The secondary structure of HpaXcm involved two α-helices in the N- and C-terminal, and the solvent accessibility values of Leu40, Leu44, Ile48, Met93, Leu94 and Val98 were 0 (Fig. 3a). In other words, these amino acid residues located in the α-helical regions are almost buried. The *a* and *d* positions of the CC structure are hydrophobic amino acids, which are responsible for the formation of the CC. The hydrophobic interactions between hydrophobic amino acids prompt the two α-helices to form supercoils^20^. The CC prediction involved two complete heptads in the N-terminal α-helical region: 33-QGISEKQ-LDQLLTQ-46, and Gln33, Ser36, Leu40, Leu43, Leu47 and Ala50 (residues at *a* and *d* positions) were key amino acids in the formation of the CC (Fig. 3b). The predicted HpaXcm tertiary structure model was more intuitive so that Leu40, Leu43, Leu47 and Ala50 were kept at the hydrophobic side of the α-helix (Fig. 3c). Hydrophobic interactions between these amino acids cause α-helices to aggregate and form oligomeric aggregates, which play an important role in protein function. Accordingly, we concluded that the CC structure of HpaXcm associated with functions is located at the protein surface.

Tobacco leaves injected with HpaXcm and HpaXcm-B produced obvious necrotic patches, which indicated the activity of the HR and the thermostability. The necrosis ratio produced by HpaXcm-B was significantly different from that of HpaXcm, suggesting that a temperature of 100 °C affects the HR activity but is not high enough to inhibit it (Fig. 4 right). The necrosis ratios of tobacco leaves treated with HpaXcm and HpaXcm-B before infection with TMV were significantly lower than that of the control leaves, which indicated that HpaXcm induces resistance to TMV in tobacco and has thermal stability (Fig. 5a). However, the necrosis rate of leaves treated with HpaXcm-B was higher than that of leaves treated with HpaXcm (Fig. 5b), suggesting that the ability of HpaXcm to induce disease resistance had been weakened by boiling HpaXcm at 100°C. The root lengths and fresh weights of plants that germinated from seeds soaked with HpaXcm and HpaXcm-B were significantly higher than that of the control group, and there was no significant difference between the HpaXcm and HpaXcm-B treatments, indicating that HpaXcm has the ability to promote root growth and that this was not affected by the 100 °C treatment (Fig. 6b). We observed that although the HR activity and disease resistance of HpaXcm was reduced at 100 °C, the 100 °C treatment had little impact on growth promotion. In previous studies, the growth-promoting of Hpa1Xoo has been reported^21,22^, and its application has been studied^23^. Thus, we chose Hpa1Xoo to provide a contrast when comparing the activity of Hpa1Xoo with that of HpaXcm. Compared with Hpa1Xoo at the same concentration, the necrosis rate induced by HpaXcm was apparently higher (Fig. 4 right), that is, the HR activity of HpaXcm is stronger than that of Hpa1Xoo. Compared with Hpa1Xoo at the same concentration, the root lengths and fresh weights of plants that germinated from seeds soaked with HpaXcm were apparently higher, that is, the growth promoting ability of HpaXcm was stronger than Hpa1Xoo (Fig. 6b), which means that HpaG-Xcm has potentially more value in terms of agricultural applications and development as a growth promoter.

The HR functional domains of HpaXcm and Hpa1Xoo are located in the N-terminal α-helical regions, and both of them have the possibility of a CC structure. The most important difference between them is that the N-terminal α-helical region of HpaXcm contains Thr and is classified as a T-type harpin, whereas Hpa1Xoo contains Cys and is classified as a C-type harpin. The hydrophobicity of Cys (which has a hydrophobic parameter of 2.5) is stronger than Thr (which has a hydrophobic parameter of −0.7) and, hence, Hpa1Xoo has stronger hydrophobic interactions. Previous studies have suggested that the HR activity of the Hpa1Xoo36-52 fragment was increased after heat treatment^22^, which is the opposite of our findings for HpaXcm. We speculate that the interactions of the CC structure probably affects the HR activity and the thermal stability of HpaXcm.

**Figure 8:**
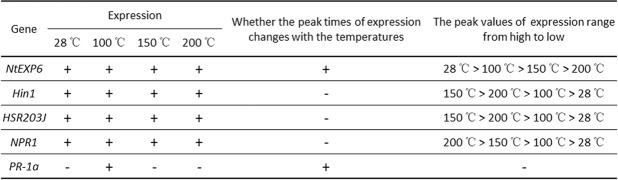
Analysis of the expression of *NtEXP6*, *Hin1*, *HSR203J*, *NPR1* and *PR-1a* in tobacco leaves induced by HpaXcm that had been heated at different temperatures: 28 °C (unheated), 100 °C, 150 °C and 200 °C.

Unlike the *PR-1a* gene, *NtEXP6*, *Hin1*, *HSR203J* and *NPR1* were significantly expressed in leaves 6 h after being subjected to the 28 °C HpaXcm, 100 °C HpaXcm or 200 °C HpaXcm treatments. All five genes were expressed in leaves after being subjected to the 150 °C HpaXcm treatment. The expression of *PR-1* is regulated by many factors, such as the accumulation of salicylic acid and the expression of *NPR1*^24^. The lack of *PR-1a* expression in leaves 6 h after being subjected to the 28 °C HpaXcm, 100 °C HpaXcm or 200 °C HpaXcm treatments is probably due to an insufficient accumulation of salicylic acid. The peak times of *NtEXP6* and *PR-1a* expression changed with heating temperature, whereas *Hin1*, *HSR203J* and *NPR1* peak expression times remained unchanged (Fig. 8). The results showed that heating HpaXcm at different temperatures had different effects on the expression of the five genes. As the protein treatment temperature increased, the expression of *NtEXP6* decreased, i.e., the higher the temperature, the lower the ability of HpaXcm to induce *NtEXP6* expression. By contrast, the higher the temperature, the stronger the ability of HpaXcm to induce *NPR1* expression. With the increase of protein treatment temperature, the expression of *Hin1* and *HSR203J* increased and then decreased, with the highest expression levels induced by the 150 °C HpaXcm treatment (Fig. 8). Protein-specific functions are determined by their specific conformations. Changes in the spatial structure of protein are accompanied by changes in biological activity. We speculate that the spatial structure of HpaXcm changes with high temperature heating, resulting in an enhanced, weakened or even a loss of the ability to induce gene expression.

In this study, we showed not only that HpaXcm has the common characteristics of a harpin protein but also that the HR activity and growth promoting effect of HpaXcm were stronger than those of Hpa1Xoo at the same concentration. We speculate that the spatial structure of HpaXcm may change when subjected to high temperatures and that the stability of interactions between CC and plants is probably associated with the HR activity and thermal stability. These findings provide a basis for further exploring the functional mechanism and heat resistance mechanism of harpins.

## Materials and Methods

### Bacteria, virus and plant materials

*Xanthomonas citri* pv. *mangiferaeindicae* HNHK and BL21/pGEX-HpaXm were maintained in the laboratory at −80 °C. Seeds of tobacco (*Nicotiana tabacum* L. cv. NC89) and *Arabidopsis thaliana*, ecotype Columbia were stored in the laboratory at 4 °C. *X*. *citri* pv. *mangiferaeindicae* strains were cultured in nutrient broth (NB) and on nutrient agar (NA) medium^25^ at 28 °C. *Escherichia coli* DH5α was cultured in Luria broth (LB) and on LB agar medium with a final concentration of 100 µg/ml ampicillin at 37 °C^6^. The leaves of *Tobacco mosaic virus* (TMV) were stored at −80 °C after rapid freezing in liquid nitrogen.

### Cloning and construction of the *hpaG-Xcm* expression vector

The *hpaG-Xcm* fragment was cloned from *X*. *citri* pv. *mangiferaeindicae* using PCR technology, the primers (Supplemental Table S1) were designed based on the *hpaXm* sequence (GenBank Accession No. DQ643828) from *X*. *citri* subsp. *malvacearum*. *X*. *citri* pv. *mangiferaeindicae* genomic DNA was extracted using the OMEGA HP Plant DNA Kit (OMEGA, Norcross, GA, USA.). The following procedure was used for the PCR reaction: pre-denaturation at 95 °C for 5 min, followed by 35 cycles of amplification (95 °C for 30 s, 58 °C for 30 s, 72 °C for 1 min), and then a final extension of 7 min at 72 °C^26^. The PCR product was purified using the OMEGA Gel Extraction Kit (OMEGA, Norcross, GA, USA), connected with the pMD18-T vector (TransGen Biotech, Beijing, China) to become T-hpaG-Xcm, and then transformed into the *E*. *coli* strain DH5α and sequenced. The T-hpaG-Xcm and pGEX-HpaXm plasmids were extracted using a Plasmid Mini Kit (OMEGA, Norcross, GA, USA). *hpaG-Xcm* and *pGEX-EF*^27^, the fragments obtained by double digestion, were connected using the *Bam*HI-*Sac*I sites, and then transformed into *E*. *coli* strain BL21 (DE3) and sequenced.

### Analysis of the primary, secondary and tertiary structures of HpaG-Xcm

Multiple alignment of protein sequences was performed using ESPript3.0 (http://espript.ibcp.fr/ESPript/cgi-bin/ESPript.cgi)^28^. A phylogenetic tree was constructed using MEGA5.0 software^4^. The conserved motifs were analysed using MEME (http://meme-suite.org/tools/meme)^29^. The homology between amino acid sequences was computed using DNAMAN software. The parameters of HpaG-Xcm were computed using the ProtParam tool (https://web.expasy.org/protparam/?_ga=1.7545010.1849019704.1487649425)^30^. The secondary structure, solvent accessibility and tertiary structure were predicted using I-TASSER (https://zhanglab.ccmb.med.umich.edu/I-TASSER/)^31,32^. The coiled-coil (CC) structure was predicted using Coiled-coil Prediction (https://npsa-prabi.ibcp.fr/cgi-bin/npsa_automat.pl?page=npsa_lupas.html)^33^. The 3D structure model was modified using PyLOM software. The amino acid sequences of homologous proteins were obtained from NCBI (https://www.ncbi.nlm.nih.gov/): HpaG-Xcm (ATB17312.1) from *X*. *citri* pv. *mangiferaeindicae*, HpaG-Xam (ATB17313.1) from *X*. *phaseoli* pv. *manihotis*, Hpa1Xoo-2 (ABU48601.1) from *X*. *oryzae* pv. *oryzae*, Hpa1Xoo (ACD56757.1) from *X*. *oryzae* pv. *oryzae*, HpaXm (ABG36696.1) from *X*. *citri* subsp. *malvacearum*, Hpa1Xoc (AAW59494.2) from *X*. *oryzae* pv. *oryzicola*, XopA-Xcv (AAL78294.1) from *X*. *campestris* pv. *vesicatoria*, HpaXcc (NP_636614.1) from *X*. *campestris* pv. *campestris*, HpaG-Xag (ABK51582.1) from *X*. *axonopodis* pv. *glycines* and Hpa1Xac (AAM35307.1) from *X*. *axonopodis* pv. *citri*.

### HpaG-Xcm protein expression, purification and western blot

LB liquid medium containing ampicillin was inoculated with BL21/pGEX-HpaXcm (pGEX-HpaG-Xcm transformed into BL21 (DE3)). Isopropyl-β-D-thiogalactoside (IPTG) with final concentrations of 0.05 mM and 0.1 mM were added to induce protein expression, until the optical density at 600 nm increased to 0.6–0.8, and then cultured at 28 °C and 37 °C for 3 h and 5 h, respectively. The bacteria collected after centrifugation were resuspended in 1 × PBS (phosphate buffered saline) (Solarbio, Beijing,China) and broken using an ultrasonic wave. After centrifugation, the supernatants and precipitates were collected and identified by performing 12% sodium dodecyl sulfate polyacrylamide gel electrophoresis (SDS-PAGE)^6,25^. Purified GST-HpaXcm (the fusion protein of a glutathione *S*-transferase (GST)-tag and HpaG-Xcm) was obtained from crude protein using a GST-tag Protein Purification Kit (Beyotime, Shanghai, China), and GST-HpaXcm was cleaved using thrombin (GE, Boston, MA, USA) at 22 °C for 16 h to obtain the purified HpaG-Xcm. HpaG-Xcm was diluted to 10 μM using PBS before determining the protein concentration by performing a bicinchoninic acid assay (BCA, Solarbio, Beijing, China). GST-HpaXcm was blotted using a polyclonal antibody against GST and a goat anti-rabbit IgG-HRP antibody.

### Hypersensitive response (HR) assays

An HR experiment was conducted on the completely unfolded leaves of 7–8-week-old tobacco plants. Proteins (10 μM) were injected into tobacco leaves, with PBS used as a negative control, using methods that have been previously described^10,34^. Scabs were observed after 2 d. The ratio of necrotic area to injection area represents morbidity. The areas of injection and necrosis were calculated using ImageJ software. Experiments were repeated three times and 10 plants were treated with each protein.

### Tobacco TMV resistance assays

The completely unfolded tobacco leaves were pretreated with proteins at concentrations of 10 μM for 16 h and then inoculated with TMV. PBS was used as a negative control^8^. The scabs were observed after 6 d. The percentage of necrotic area to leaf area represents morbidity. The areas of injection and necrosis were calculated using ImageJ software. Experiments were repeated three times and 10 plants were treated with each protein.

### Root growth promotion assays

*A*. *thaliana* seeds were soaked in protein solutions (10 μM) at 28 °C for 6 h, and then transferred to MS agar medium. The plates were then placed vertically in a 24 °C incubator with a 10 h night cycle^21,22^. Seeds soaked in PBS were used as a negative control. The root length and the fresh weight of the germinated seeds were recorded after 15 d. Experiments were repeated three times and 30 seeds were treated with each protein.

### qRT-PCR

HpaG-Xcm proteins (10 μM) were pretreated for 10 min at 28 °C, 100 °C, 150 °C, or 200 °C and then cooled to room temperature before injecting the proteins into whole tobacco leaves. Leaves were collected at 0, 1, 3 and 6 h; the leaves collected at 0 h were used as a control. The RNA of leaves subjected to the different treatments was extracted using an RNAprep Pure Plant Kit (TIANGEN, Beijing, China) and TransScript One-Step gDNA Removal and cDNA Synthesis SuperMix (TRANS, Beijing, China) was used to reverse transcript cDNA. The relative expression of the HR-related genes *Hin1*^16^ and *HSR203J*^17^, the defence-related genes *NPR1* and *PR-1a*^11^, and the growth promotion-related gene *NtEXP6*^18^ were detected by quantitative real-time PCR (qRT-PCR) using SuperReal PreMix Plus (SYBR Green) (TRANS, China). The relative expression of genes was calculated using the 2^−ΔΔCT^ formula with *EF-1a* as a control for normalization. The following procedure was used for the PCR reaction: pre-denaturation at 95 °C for 10 min, followed by 40 cycles of amplification (95 °C for 10 s, 60 °C for 35 s, 72 °C for 20 s)^22^. The primers are listed in Supplemental Table S1. Experiments were repeated three times and 10 plants were treated with each protein.

### Data processing

All data were counted and analysed using SPSS software. The values shown in the results were the means and standard deviations (SD) of three repetitions. Significant differences were analysed using multiple comparisons (LSD method, *P < 0.05, **P < 0.01).

## Supplementary information

High temperatures affect the hypersensitive reaction, disease resistance and gene expression induced by a novel harpin HpaG-Xcm
