# Supplementary material for: High temperatures affect the hypersensitive reaction, disease resistance and gene expression induced by a novel harpin HpaG-Xcm
Source: Sci Rep. 2019 Jan 30;9:990. doi: 10.1038/s41598-018-37886-9 (PMC6353989; doi:10.1038/s41598-018-37886-9)
Supplement: Supplementary file 1 — High temperatures affect the hypersensitive reaction, disease resistance and gene expression induced by a novel harpin HpaG-Xcm [file 41598_2018_37886_MOESM1_ESM.pdf]

Supplemental Information

High temperatures affect the hypersensitive reaction, disease resistance and gene expression induced by a novel harpin HpaG-Xcm

Xiaoyun Zhou, Yue Liu, Jiamin Huang, Qinghuan Liu, Jianzhang Sun, Xinfeng Cai, Peng Tang, Wenbo Liu & Weiguo Miao

Supplemental Table

Table S1. Primers used for the amplification of HpaG-Xcm and qRT-PCR

Supplemental figures

Figure S1. The expression of GST-HpaXcm (fusion protein GST-HpaG-Xcm) and HR elicited by GST-HpaXcm induced under different conditions.
Figure S2. SDS-PAGE and western blot analysis of the expressed fusion protein GST-HpaXcm and purified HpaXcm.
Figure S3. The western blot analysis for GST-HpaXcm cloned in pGEX-EF.

Table S1. Primers used for the amplification of HpaG-Xcm and qRT-PCR

**Primer**	**Primer sequence (5'-3')**	**Description**
HpaG-Xcm-F	GGATCCAGTTAATCAGAGAGGAATCGTCATG	PCR &sequencing *hpaG-Xcm*
HpaG-Xcm-R	GAGCTCGGTAGGGGCGACCAACAGTTCGTTA	
EF1a-F	ATCAATCCAGGTCATCATCA	RT-qPCR
EF1a-R	AAGTTCCTTACCAGAACGCC	
HSR203J-F	AGCTATGAAAAAGGGGGAAA	RT-qPCR
HSR203J-R	AACCATTAGAACGTGACAATC	
Hin1-F	TGACTATTAGAAACCCCAACA	RT-qPCR
Hin1-R	CTTCCATCTCATAAACCCCT	
PR-1a-F	AACCTTTGACCTGGGACGAC	RT-qPCR
PR-1a-R	CAACACGAACCGAGTTACGC	
NtEXP6-F	CCCTGTTGTTTATCGTCG	RT-qPCR
NtEXP6-R	GTTTGCCAGTTCTGACCC	
NPR1-F	TCTGTATCTCTTGCTATGGC	RT-qPCR
NPR1-R	TGCTTCTTCAGTTGACGCTC	

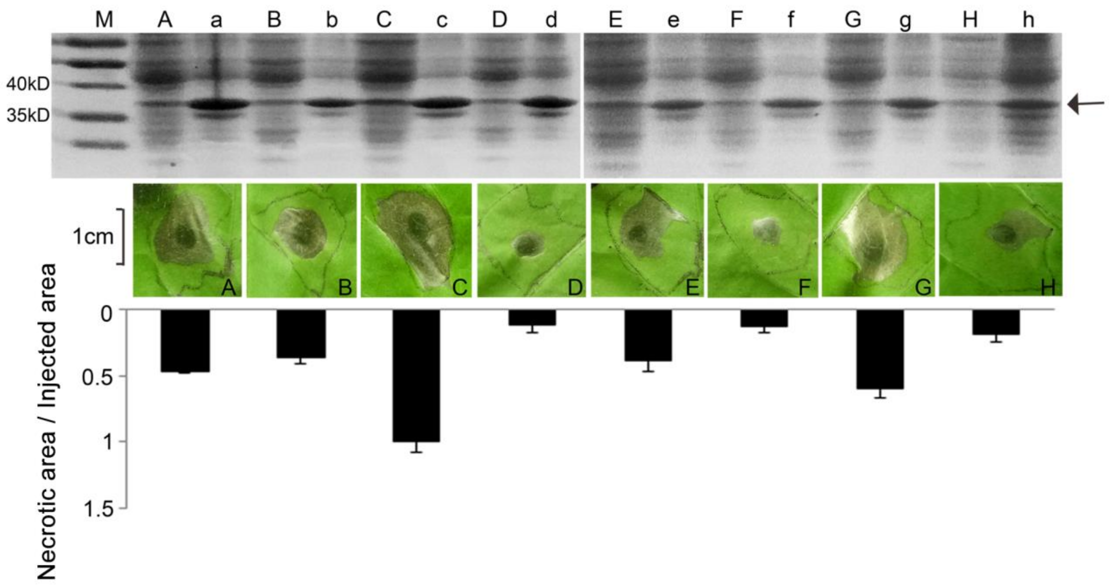

Figure S1. The expression of GST-HpaXcm (fusion protein GST-HpaG-Xcm) and HR elicited by GST-HpaXcm induced under different conditions. (**A–H**) Soluble proteins; (**a-h**) insoluble proteins; (**A, a**) were induced with 0.05 mM isopropyl-β-D-thiogalactoside (IPTG) at 28 °C for 5 h; (**B, b**) were induced with 0.1 mM IPTG at 37 °C for 5 h; (**C, c**) were induced with 0.1 mM IPTG at 28 °C for 5 h; (**D, d**) were induced with 0.1 mM IPTG at 37 °C for 3 h; (**E, e**) were induced with 0.05 mM IPTG at 37 °C for 3 h; (**F, f**) were induced with 0.05 mM IPTG at 28 °C for 3 h; (**G, g**) were induced with 0.05 mM IPTG at 37 °C for 3 h; (**H, h**) were induced with 0.1 mM IPTG at 28 °C for 3 h; (**M**) protein marker. The arrow indicates the specified band. Tobacco leaves were injected with supernatants at a concentration of 10 μM. Necrotic lesions were observed after 2 days. The scale bar represents 1 cm. The histogram expresses the ratio of the necrotic area to the injected area. Error bars indicate the standard error of the mean (n = 3). Lanes A, a, B, b, C, c, D and d were in the same gel. Lanes E, e, F, f, G, g, H and h were in the same gel.

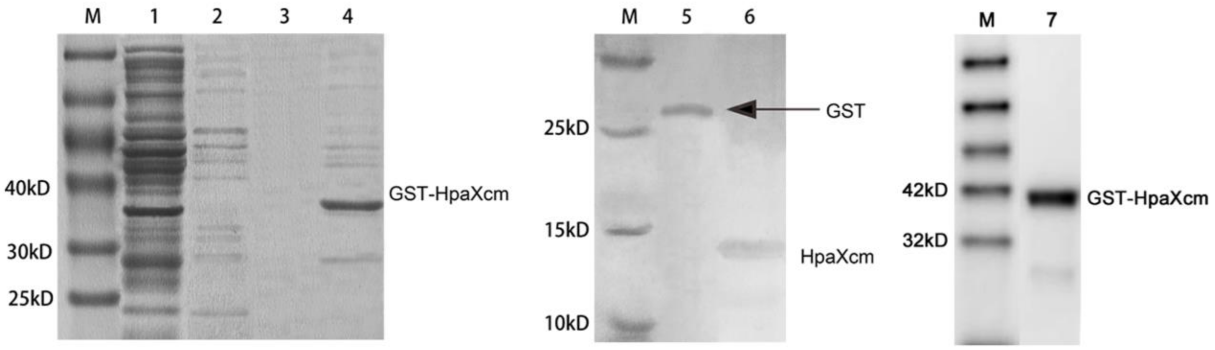

Figure S2. SDS-PAGE and western blot analysis of the expressed fusion protein GST-HpaXcm and purified HpaXcm. (**Lane 1**) the supernatants of BL21/pGEX-hpaXcm sonicated; (**Lane 2**) flow through; (**Lane 3**) phosphate buffered saline (PBS); (**Lane 4**) purified GST-HpaXcm; (**Lane 5**) purified glutathione S-transferase (GST) from E. coli BL21 (DE3)/pGEX-HpaXcm; (**Lane 6**) purified HpaXcm; (**Lane 7**) western blot of GST-hpaXcm; (**M**) protein markers. Blots of the supernatants of BL21/pGEX-hpaXcm sonicated, flow through, PBS and purified GST-HpaXcm were run in the same gel. Blots of the purified GST from E. coli BL21 (DE3)/pGEX-HpaXcm and purified HpaXcm were run in the same gel. The full-length blots are shown in Supplementary Figure S3.

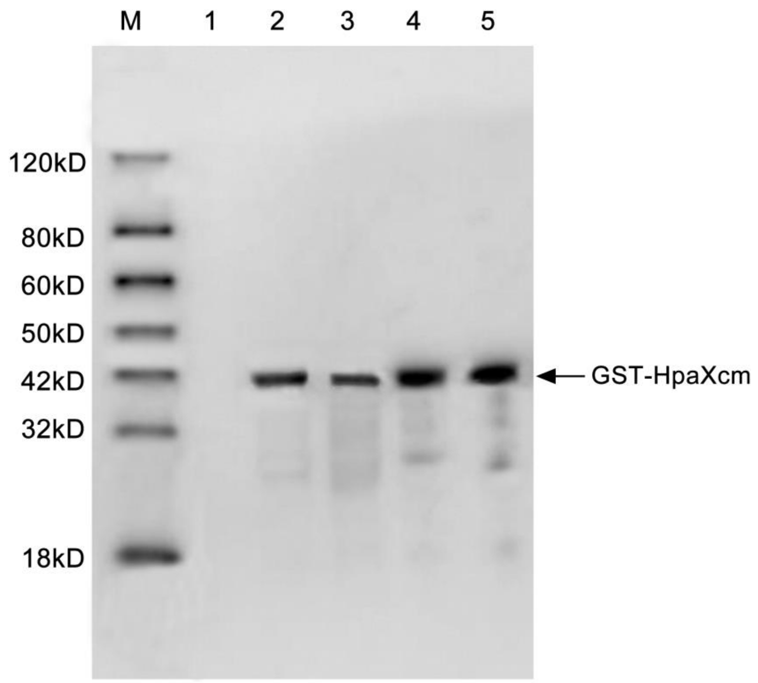

Figure S3. The western blot analysis for GST-HpaXcm cloned in pGEX-EF. (**Lane 1**) cell lysate without induction; (**Lane 2, 3**) debris of cell lysate with induction for 5 h at 28℃; (**Lane 4, 5**) supernatant of cell lysate with induction for 5 h at 28℃; (**M**) protein marker.
